# Recent advancement in OMICS approaches to enhance abiotic stress tolerance in legumes

**DOI:** 10.3389/fpls.2022.952759

**Published:** 2022-09-28

**Authors:** Amjad Ali, Muhammad Tanveer Altaf, Muhammad Azhar Nadeem, Tolga Karaköy, Adnan Noor Shah, Hajra Azeem, Faheem Shehzad Baloch, Nurettin Baran, Tajamul Hussain, Saowapa Duangpan, Muhammad Aasim, Kyung-Hwan Boo, Nader R. Abdelsalam, Mohamed E. Hasan, Yong Suk Chung

**Affiliations:** ^1^Faculty of Agricultural Sciences and Technologies, Sivas University of Science and Technology, Sivas, Turkey; ^2^Department of Agricultural Engineering, Khwaja Fareed University of Engineering and Information Technology, Rahim Yar Khan, Pakistan; ^3^Department of Plant Pathology, Faculty of Agricultural Sciences & Technology, Bahauddin Zakariya University, Multan, Pakistan; ^4^Bitkisel Uretim ve Teknolojileri Bolumu, Uygulamali Bilimler Faku Itesi, Mus Alparslan Universitesi, Mus, Turkey; ^5^Laboratory of Plant Breeding and Climate Resilient Agriculture, Agricultural Innovation and Management Division, Faculty of Natural Resources, Prince of Songkla University, Hat Yai, Thailand; ^6^Subtropical/Tropical Organism Gene Bank, Department of Biotechnology, College of Applied Life Science, Jeju National University, Jeju, South Korea; ^7^Agricultural Botany Department, Faculty of Agriculture (Saba Basha), Alexandria University, Alexandria, Egypt; ^8^Bioinformatics Department, Genetic Engineering and Biotechnology Research Institute, University of Sadat City, Sadat City, Egypt; ^9^Department of Plant Resources and Environment, Jeju National University, Jeju, South Korea

**Keywords:** legumes, climate change, drought stress, marker-assisted breeding, marker-trait association

## Abstract

The world is facing rapid climate change and a fast-growing global population. It is believed that the world population will be 9.7 billion in 2050. However, recent agriculture production is not enough to feed the current population of 7.9 billion people, which is causing a huge hunger problem. Therefore, feeding the 9.7 billion population in 2050 will be a huge target. Climate change is becoming a huge threat to global agricultural production, and it is expected to become the worst threat to it in the upcoming years. Keeping this in view, it is very important to breed climate-resilient plants. Legumes are considered an important pillar of the agriculture production system and a great source of high-quality protein, minerals, and vitamins. During the last two decades, advancements in OMICs technology revolutionized plant breeding and emerged as a crop-saving tool in wake of the climate change. Various OMICs approaches like Next-Generation sequencing (NGS), Transcriptomics, Proteomics, and Metabolomics have been used in legumes under abiotic stresses. The scientific community successfully utilized these platforms and investigated the Quantitative Trait Loci (QTL), linked markers through genome-wide association studies, and developed KASP markers that can be helpful for the marker-assisted breeding of legumes. Gene-editing techniques have been successfully proven for soybean, cowpea, chickpea, and model legumes such as *Medicago truncatula* and *Lotus japonicus*. A number of efforts have been made to perform gene editing in legumes. Moreover, the scientific community did a great job of identifying various genes involved in the metabolic pathways and utilizing the resulted information in the development of climate-resilient legume cultivars at a rapid pace. Keeping in view, this review highlights the contribution of OMICs approaches to abiotic stresses in legumes. We envisage that the presented information will be helpful for the scientific community to develop climate-resilient legume cultivars.

## Introduction

**Figure 1:**
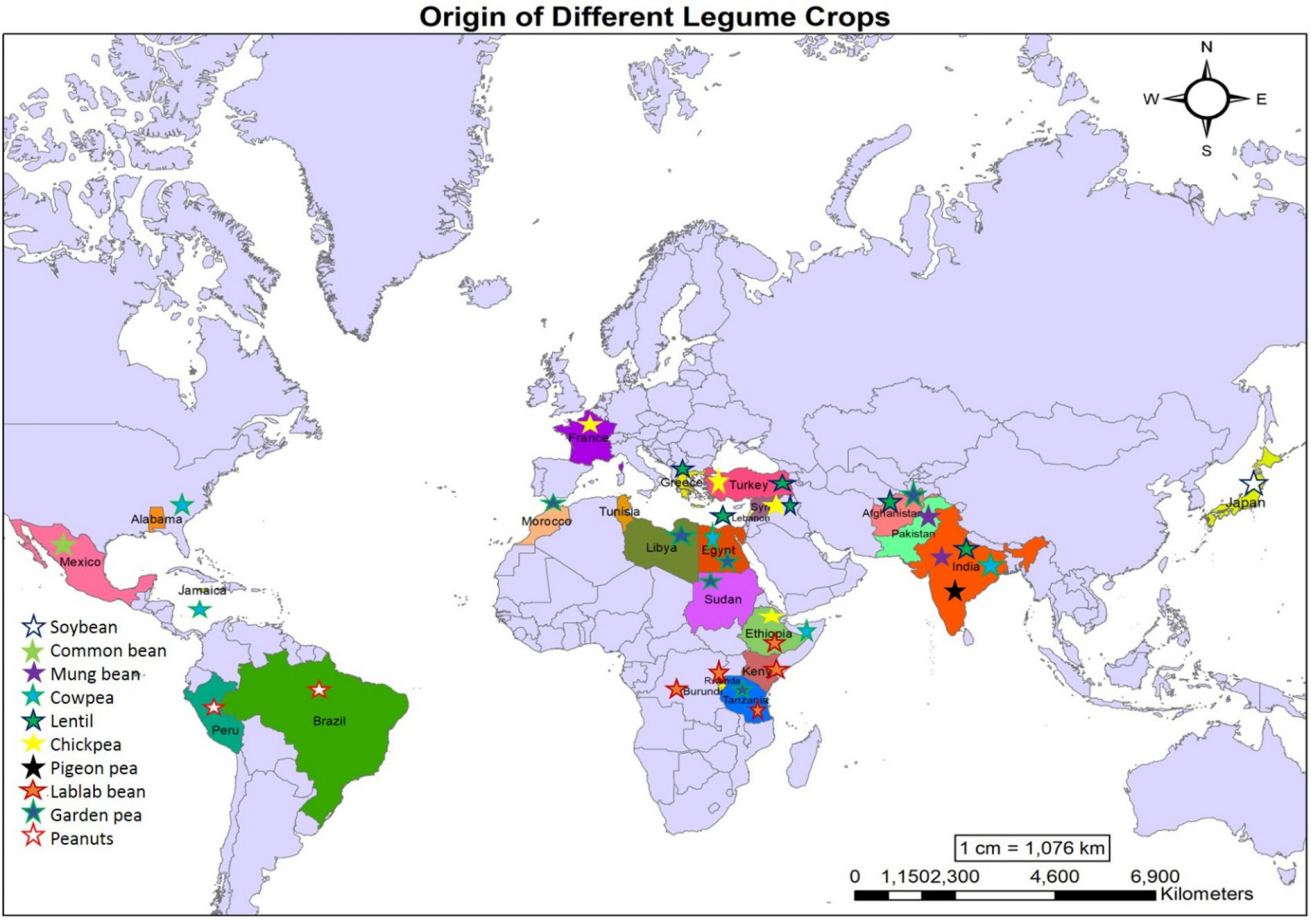
Origin centers of various legume crops.

The grand challenge facing scientists in the twenty-first century is to optimize climate change adaptation, agricultural productivity, food security, and environmental protection. Climate-related changes will likely boost the severity of both sole and joint abiotic stresses, especially drought, cold, salinity, and heat (Pandey et al., 2017; Anwar et al., 2022). These climate-change vulnerabilities pose a high threat to global food and nutritional security. Crops that can help overcome the effects of climate change are urgently needed. Legumes may prove to be one such suitable group of crops with promising future possibilities. Legumes being the members of Fabaceae family have a great value for their versatile usage and demand for humans and livestock. Legumes are a staple food for millions of people all over the world and serve as a cheap source of high-quality protein. As a result of its higher global consumption, this family ranks second after Gramineae and is the third largest flowering plant family followed by Asteraceae and Orchidaceae (Çakir et al., 2019). Presently, 88% of species of these families are examined and 750 genera consisting of 17,000–18,000 species play a significant role as food grains, pasture, and agroforestry to maintain the environments (Gupta and Pandey, 2021). Legumes are nutritionally used as staples worldwide. Legumes are a low-cost and good source of vitamins, carbohydrates, protein, and fiber. Concerning global distribution, chickpeas, pigeon pea, and lentils are widely cultivated in South Asian countries; faba beans are primarily cultivated in North Africa, China, and the Mediterranean countries; cowpeas in West African (WA) countries; soybean and other oilseed crops predominantly found in Indonesia, China and Japan while the Central and South America (C&SA) is also famous for the cultivations of beans (Joshi and Rao, 2017). The origins of legume crops are shown in Figure 1.

The consumption of legumes in different forms is common, with a beneficial aspect for the human diet, aiding good health. Legumes are full of substantial elements of diets including necessary proteins, minerals, complex carbohydrates, amino acids, dietary fibers, and vitamins such as riboflavin, vitamins B complex, tocopherols, pyridoxine, thiamine, and folates (Mefleh et al., 2022). Phaseolin is the major globulin in domesticated *Phaseolus* beans. Phaseolin is a protein containing a neutral sugar, usually mannan, and it is organized into three subunits, each of which possesses different molecular weights, isoelectric points, and glycosylation degrees. The legumes contain low saturated fats, and for that reason can be considered cholesterol-free. Protein is the main element for good health and is found high in legumes (40%) as compared to cereals (17%) (Kamau et al., 2020). Legumes seeds contain manyproteins such as oligomeric globulins, albumins, glutelins, and prolamins. These proteins are generally synthesized in the seed, and provide support for seedling germination. The amino acid analysis of legumes revealed a moderate quantity of sulfur-containing L-tryptophan and amino acids but high content of the primary Lysine. The carbohydrate is present in the form of starch, galactose ribose, á-galactosides, glucose, maltose, sucrose, and fructose as well as fermentable fibers in legumes seeds, and plants. The presences of amylopectin and amylose in dry grains and lignin, oligosaccharides, hemicellulose, cellulose, and pectin in plants are a vital source of carbohydrates that help in digestion. A significant amount of micronutrients is also present in legume crops. The micronutrients such as calcium, potassium, selenium, iron, magnesium, copper, and zinc are reported in mung bean, common bean, faba beans, chickpeas, lupins, and lentils which collectively play a vital role in body development (Roorkiwal et al., 2021). The nutritional comparison among food legumes is given in Table 1.

**Table 1:** Nutritional comparison among food legumes.

**Crops**	**Cowpea**	**Chickpea**	**Lentil**	**Mung bean**	**Kidney bean**	**Pigeon pea**	**Soybean**	**Moth-bean**
Protein (%)	24	21	25	24	24	22	37	23
Dietary fiber (%)	11	12	11	16	25	15	9	–
Carbohydrate (%)	60	63	63	63	60	63	30	62
Lipids (%)	2	6	1	1	1	2	20	2
K (μg g^−1^)	13,750	7,180	6,770	12,460	14,060	13,920	17,970	11,910
Zn (μg g^−1^)	61	28	33	27	51	28	50	19
Ca (μg g^−1^)	850	570	350	1,320	1,430	1,300	2,770	1,500
Na (μg g^−1^)	80	240	60	150	240	170	20	300
Fe (μg g^−1^)	100	43	65	67	82	52	157	109
P (μg g^−1^)	4,380	2,520	2,810	3,670	4,070	3,670	7,040	4,890
Mg (μg g^−1^)	3,330	790	470	1,890	1,400	1,830	2,800	3,810
Vitamin A (IU)	33	67	39	114	0	28	22	32
Vitamin C (μg g^−1^)	15	40	45	48	45	0	60	40
vitamin B6 (μg g^−1^)	4	5	4	4	4	3	4	4
Vitamin E (μg g^−1^)	0	8	5	5	2	0	9	0
Thiamin (μg g^−1^)	7	5	9	6	5	6	9	6

Despite their commercial and economic importance, legumes have not received the same level of attention as cereals to increase crop productivity. The legumes crop is threatened by a series of biotic diseases especially caused by fungi and nematodes. The fungal diseases such as ascochyta blight (*Ascochyta rabiei*), charcoal rot (*Macrophomina phaseolina*), powdery mildew (*Erysiphcpoiygoni*), alternaria blight (*Alternaria alternate*), botrytis gray mold (*Botrytis cinerea*), cercospora leaf spot (*Cercospora canescens*), collar rot (*Sclerotium rolfsii*), phytophthora blight (*Phytophthora drechsleri*), stemphylium blight (*Stemphylium* sp), fusarium wilt (*Fusarium oxysporum* f. sp. *ciceris*), wet root rot (*Rhizoctonia solani*), vascular wilt (*F. oxysporum* f. sp. *lentis*) are contributed to major yield loss in legumes crops as compared to all others (Sampaio et al., 2020; Mahmoud, 2021; Pandey and Basandrai, 2021; Urva, 2021). Nematodes (Cyst and root-knot) are also a major factor of yield loss in legumes (Zwart et al., 2019). Similarly, crops are susceptible to insects, weeds, and diseases, which are also responsible for devastating yield losses (Reddy et al., 2004).

Globally, 55 developing countries having semiarid and tropical areas are affected by abiotic stress where mainly cultivation consists of grain legumes. In abiotic stress, multiple stresses are involved like drought, waterlogging, chilling, salinity, and high temperature are the main cause of the decrease in crop production annually (Ozturk Gokce et al., 2020). The cultivated land faced about 90% environmental stresses. Temperature plays a significant role in the survival of plant and their growth along with root nodules development. The rise in high-temperature result in poor pollination, and cause a significant reduction in the crop yield (Settele et al., 2016). Drought is an important abiotic stress issue that terribly affects crop production. Drought hinders the legume symbiotic routine, slowing down the growth, and eventually leading to decreased crop yield (Anjum et al., 2017). However, the influence of drought on the exposed plant is mainly dependent on the long drought period and magnitude of the stress. Cold-induced stress negatively affects photosynthetic pigments and capacity, antioxidant defense mechanisms, and mineral nutrient uptake; it alters metabolic and hormonal processes, and it disturbs plant developmental stages from seed germination to maturation. Plants exposed to low temperatures can increase the accumulation of reactive oxygen species (ROS), including superoxide anion radicals (${\text{O}}_{2}^{-•}$), singlet oxygen (_1_O^2^), hydrogen peroxide (H_2_O_2_), and hydroxyl ions (OH^−^), which damage mitochondria and chloroplasts and can lead to cell death (Gill and Tuteja, 2010). Salinity is also an important abiotic stress issue that is proved as harmful to the survival of legumes (Shabala and Munns, 2012). In newly emerging leaves, the abscisic acid (ABA) level rises in the transpiration portion due to sodium chloride (Badhan et al., 2018). In the leaf veins and root, an ATP binding cassette (ABC) carrier was expressed that play a fundamental role toward ABC by showing hypersensitivity in plants regarding seedling and germination phase. Genetically designed plants were upregulated for ABA expression from separated leave have displayed a reduction in water loss as well as higher lead temperature than the normal plants (Raza et al., 2019).

Enhancement of already existing germplasm through focused evolution needs tracking of the desired traits to combine them. Previously, phenotype assisted in tracking the qualities of a plant in better ways that are nowadays exchanged in more meaningful ways in sense of DNA markers. Combinations of molecular studies and other breeding tools have moved into deeper knowledge to study the possible traits of a selected plant, including genomic and gene regulatory traits (Dhaliwal et al., 2020). The whole bunch of genes quantitative trait loci (QTLs) are studied along with the molecular markers that are connected with traits for trustworthy marker-aided breeding. The marker technology development in legumes is relatively slower as compared to cereals, giving them the label of an orphan crop (Gresta et al., 2016). The gradual development of molecular markers proceeds includes first-generation markers i.e., Restriction fragment length polymorphism (RFLP), amplified fragment length polymorphism (AFLP), random amplification of polymorphic DNA (RAPD), producing data of various loci in a single turn, mainly used in the study of diversity in pigeon pea, winged bean, cowpea, cluster bean, and dolichos bean (Chapman, 2015; Kumar et al., 2020). Furthermore, sequence-oriented markers, specifically, single nucleotide polymorphism (SNPs), simple sequence repeats (SSRs), and their alternation to increase reproducibility and reliability, efficiently working in trait mapping, fine mapping as well as linkage mapping studies (Choi, 2019) have also been documented. Simple sequence repeats (SSRs)markers have been used for the assessment of genetic diversity.

SSR markers have been used in cluster bean, pigeon pea, cowpea, and winged bean, whereas inter-species SSRs have been employed in lablab for the assessment of diversity and other purposes. SSRs markers have also been used in many dolichos bean studies for the assessment of genetic diversity (Keerthi et al., 2018; Singh et al., 2021). SNPs are desired markers due to their pervasive nature and lavishness in the genetic material. SNPs have been recognized as effective and used in genetic material-aided breeding of cluster bean, pigeon pea, winged bean, cowpea, and dolichos bean. As the desired markers are recognized, it provides us a pathway for mapping traits of concern going toward cloning as well as fine mapping (Bohra et al., 2019; Nawaz and Chung, 2020). This review article will provide a brief overview of multi-omics techniques and their uses for abiotic stress research on legumes.

### Legumes genetic resources

Genetic diversity is essential for fulfilling the basic dietary and nutritional needs of an ever-increasing population, and it also serves as the foundation for selection response. Information on crop genetic resources, including pulse crops conserved in national and international gene banks, is available online at global portals such as Genesys (https://www.genesys-pgr.org/) and GRIN (https://www.ars-grin.gov/), as well as in a 1996 FAO publication titled “The second report on the state of the world's plant genetic resources for food and agriculture” (https://www.fao.org/3/i1500e/i1500e00.htm). According to the FAO report, CG centers hold 35,891 common bean accessions, 33,359 chickpea accessions, 15,588 cowpea accessions, 13,289 pigeonpea accessions, 10,864 lentil accessions, 9,186 faba bean accessions, 6,129 field pea accessions, and 3,225 grass pea accessions in trust (Table 2). Since then, the ICARDA gene bank has grown to include 50,968 accessions of pulse crops, including 15,749 chickpeas, 14,597 lentils, 10,034 faba beans, 6,131 peas, and 4,457 grass peas. Similarly, ICRISAT holds 20,764 and 13,783 accessions of chickpea and pigeonpea, respectively. CIAT, IITA, and AVRDC hold 37,938, 16,460, and 10,946 accessions of Phaseolus beans, cowpea, and Vigna species, respectively. To facilitate accessibility and better use of the germplasm available with gene banks, various sets of core (Frankel, 1984; Brown, 1989), mini-core (Upadhyaya and Ortiz, 2001), focused identification of germplasm strategy (FIGS) (Mackay et al., 2004), and reference (Odong et al., 2011) germplasm have been developed. The FIGS strategy (https://www.icarda.org/research/innovations/focused-identification-germplasm-strategy-figs) is being pursued at ICARDA, which has proved effective for various adaptive traits such as heat, drought, cold, and salt tolerance, as well as insect pest and disease resistance. FIGS sets for chickpea, lentils, grass pea, and faba bean have recently been released, which can be used to find and apply valuable genes in preferred agronomic environments. Similarly, ICRISAT-developed mini-core sets of chickpea, pigeonpea, and groundnut provide a rich source of variety for desired traits in breeding operations (Upadhyaya et al., 2013). Except for a few features, the present germplasm of pulse crops contains sufficient variety for crucial economic traits. The creation of structured and representative collections of germplasm from the worldwide collection increases the efficiency with which beneficial alleles/traits can be identified and used.

**Table 2:** Legume germplasm holdings in major gene banks.

**Crop**	**Global status**	**CG center**	**USDA**	**NBPGR,/India**
Chickpea	98,285	33,359	7,000	14,704
Lentil	58,405	10,864	–	9,989
Vigna species	–	–	–	5,549
Common bean	261,963	35,891	–	1,514
Grass pea	26,066	3,225	–	2,797
Field pea	94,001	6,129	6,161	3,070
Cowpea	65,323	15,588	1,287	3,317
Pigeonpea	40,820	13,289	4,806	12,859
Faba bean	43,695	9,186	–	–
Others	183,078	13,690	–	19,579
Total	1,069,897	141,221		73,378

CG, Crop Genebanks; NBPGR, National Bureau of Plant Genetic Resources, India; USDA, United States Department of Agriculture.

## OMICS approaches in the technological era

**Figure 2:**
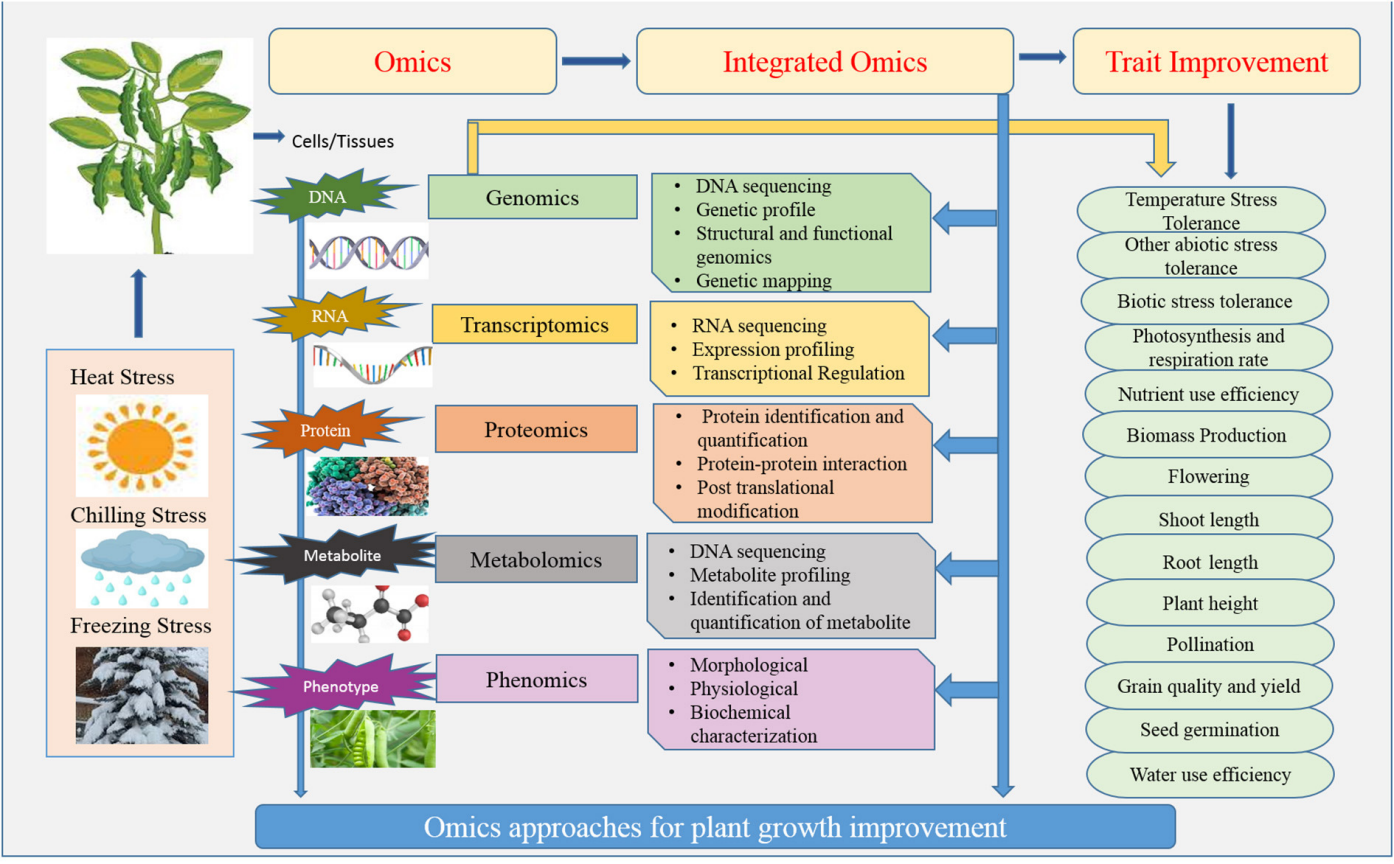
Schematic visualizing the contribution of OMIC's approaches for legume plant improvement.

Omics is a fast-evolving technique that provides the methodology, technological proficiency, and interdisciplinary and transdisciplinary advancement needed to improve awareness and identification of all the plant's genomic and transcriptomic processes. Omics is a beneficial aid for understanding the change in plant metabolism as a consequence of contact with the external environment (Kumar et al., 2021). Transcriptomic, metabolomic, and genomic tools scan and give gene - expression and protein expression levels immediately, so in the advanced era these play a vital role in plant improvements (Jha et al., 2020). Omics technology not just increases our awareness, but also gives us new insights into how plants react when stressed. Whenever, a small change occurs in genetics, nutrients, or fluctuation in the external environment, a wide range of technologies is used to perceive these variations in plants. In the recent and modern era, these advanced tools are providing support to understand the whole plant genome, and the biggest example of this is *Arabidopsis thaliana*. Innovation in the modern era started with the complete genome sequencing of this model plant. Rice, maize, and soybeans are among the main crop plants whose genomes are complicated but have been fully sequenced. The introduction of high-throughput “Omics” methodologies has ushered in a time of successful plant molecular tools for responding to environmental changes. Rapid innovations and advancements in “omics” regarding the post-genomic epoch such as molecular characterization, next-generation sequencing, modeling of various molecular and physiological knowledge, and association of these assertions with plant establishment have contributed to a successful transition to resilience and efficiency under abiotic stress conditions (Pandey et al., 2021). With the invention of next-generation sequencing techniques, it become feasible and reliable to sequence plant species. Furthermore, current omics tools such as proteomics and transcriptomics aids to understand the level of genes and protein. Different results from reported studies showed that not all the genes are turned on or off at the same time; for that reason, the metabolism adopts a complex phenotype that cannot be determined by genotype (Jha et al., 2019; Singh et al., 2020). As a result, the successful integration of genomics, transcriptomics, phonemics, proteomics, epigenomics, metabolomics, interact omics, and ionomics would aid breeders in identifying promising candidates' genes and optimal features for generating and improving the productivity of legume crops under abiotic stresses (Kumar et al., 2022). Figure 2 is showing different omics approaches for the improvement of plant growth.

## Contribution of OMICs approaches in legumes for abiotic stresses

**Figure 3:**
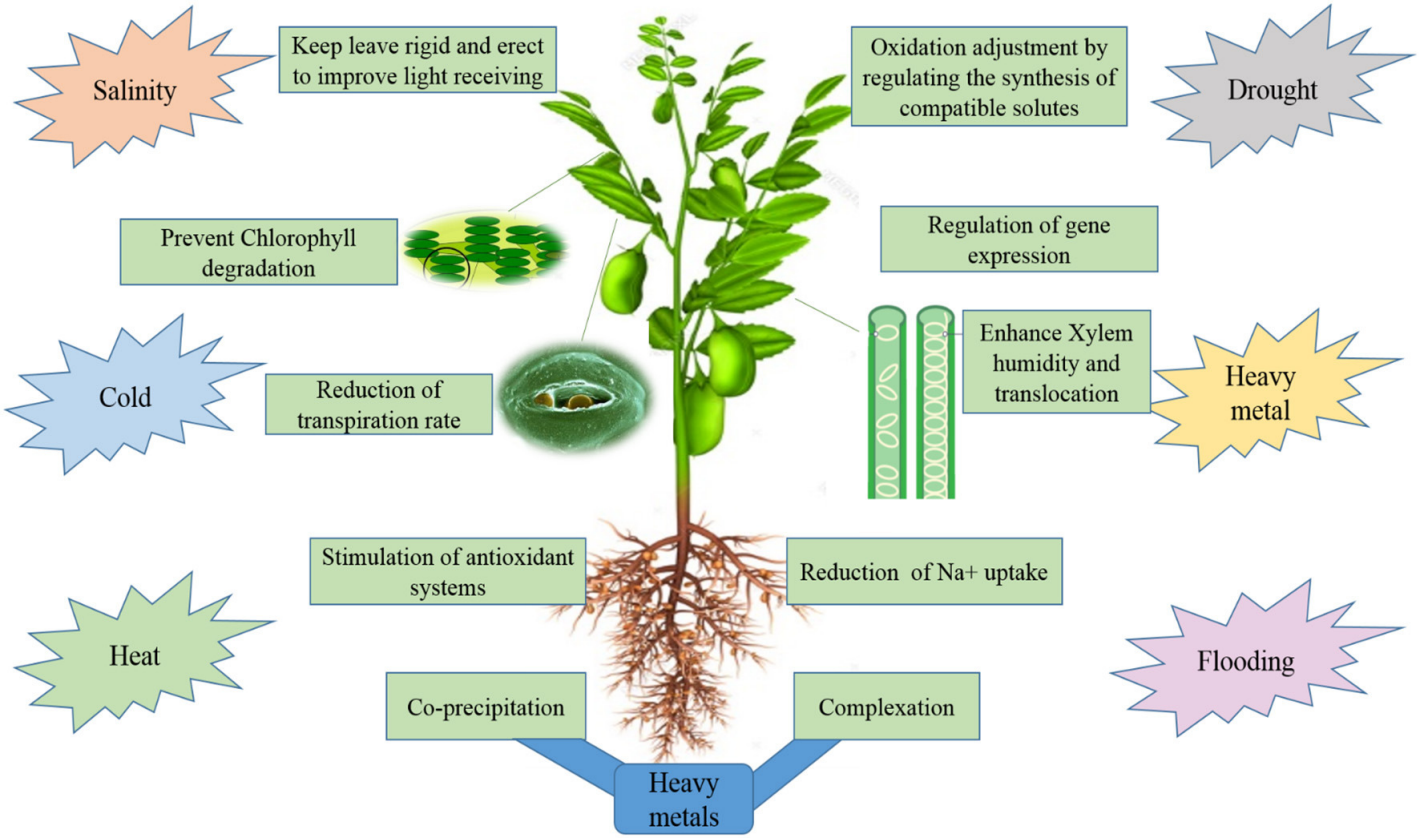
Exploring the effect of abiotic stresses on the growth of legume crops.

**Figure 4:**
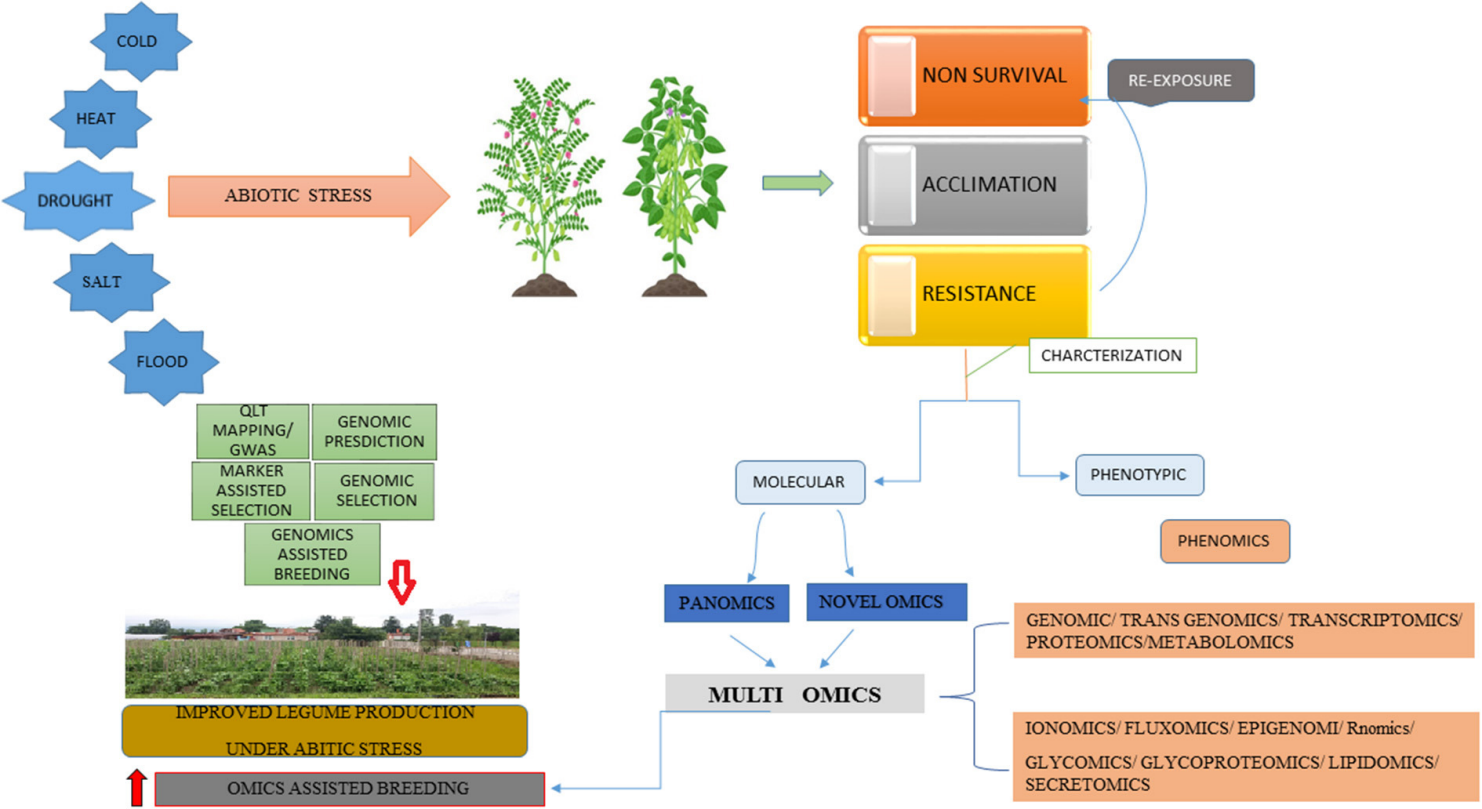
Improved abiotic stress tolerance in legumes using integrated multi-omics techniques.

The genome size of the legumes is large and some of them are polyploidy (Stai et al., 2019). As a result, model systems have been established to better recognize the legume biology and their modeling system that is developed for genetics studies regarding nodule formation and some dynamic processes like active tolerance, resistance, and susceptibility toward numerous stresses. Figure 3 provide a brief information about the effects of various a biotic stress on the growth of legumes. The basic research on legumes is performed on plant model systems, most frequently *Lotus japonicas* and *Medicago truncatula*, because of their diploid small genome, short life cycle, and enormous production of seed (Cook, 1999; Cervantes et al., 2019). The implementation of all these models has significantly enhanced the genetic and genomic datasets regarding legumes, and the similarity between distinct legume genomes has indeed augmented the data's usefulness (Stracke et al., 2004). In addition, the genome sequencing of soybean and their higher similarity to model legume species may greatly assist many genetic methods for investigation, like positional cloning. The genome sequence data is extremely valuable and serve as a great initial point, it is insufficient to understand gene activity, and the numerous metabolic reactions and regulatory responses get stimulated as the stress condition prolongs in the legumes. New suitable approaches and methods for overcoming these barriers to biotic stresses including the study of quantitative and qualitative traits are essential for checking gene expression (Wan et al., 2017). These must be done through the use of transcriptome, proteomic, and metabolomic levels study with an understanding of the use of genetically modifying crops and marker-assisted screening (Dita et al., 2006). Genomic, metabolomics, transcriptomics, transgenomics, proteomics, phenomics, and functional genomics fall under 'omics' and are admired as omics tools. Significant advancements within those distinct omics (Figure 4 and Table 3) have played an important role in the better understanding of stress responses at the molecular as well as genetic levels (Langridge and Fleury, 2011).

**Table 3:** Novel omics approaches for legumes breeding.

**Legumes**	**Abiotic stress**	**Omics technology**	**Details**	**References**
Cowpea	Drought	Phosphoproteomics	Protein phosphorylation is induced by a gradual water deficiency.	Subba et al., 2013
		Secretomics	Dehydration, the stress-responsive secretome, and the highly complex metabolic network activity in the extracellular matrix have all been studied in depth.	Gupta et al., 2015
	Oxidative	Secretomics	CaFer1's role in iron buffering and oxidative stress adaption under different weather conditions.	Parveen et al., 2016
Common bean	Chlorpyrifos	Lipidomics	Triacylglycerol levels in pods and seeds are decreasing.	Fernandes et al., 2018
Soybean	Heat	Lipidomics	Reduced expression of fatty acid desaturase results in lower quantities of lipids with 18:3 acyl chains.	Narayanan et al., 2020
	Low phosphorus	Lipidomics	Under low-phosphorus circumstances, lipid remodeling occurs.	Okazaki et al., 2017
	Flooding	Phosphoproteomics	During flood stress, the ethylene signaling pathway was critical for protein phosphorylation in root tips.	Yin et al., 2014
		Glycoproteomics	Protein N-glycosylation was negatively affected by flooding.	Showalter et al., 2016

## Genomics advances for abiotic stress tolerance in legumes

Genomics focuses on the physical integrity of the genome, with the goal of recognizing, diagnosing, and regulating genomic features throughout the chromosomes. Now we deliberate some genomic advancements to apprehend the abiotic stress endurance in legumes (Chandrashekharaiah et al., 2021).

### Molecular marker resources

The advent of genomic research has opened up new possibilities for genetic improvement of complex traits like salinity and drought endurance. In comparison to traditional breeding, a combination of genomic techniques and MAS can contribute to the identification of individual-specific genes in breeding populations at a considerably faster rate (Saade et al., 2016). SNPs are excellent for distinguishing complex traits employing massively multiplexed marker oligonucleotides like the Affymetrix GeneChip, as these are fast, high-throughput, co-dominant, highly abundant, cost-effective, and sequence-tagged (Missanga et al., 2021; Thudi et al., 2021). High throughput Axiom® SNP array genotyping is an efficient and cost-effective method for genotyping and the development of high-density linkage maps. Recently “Axiom® *CicerSNP* Array” has been developed for genotyping of Recombinant inbred lines of chickpea (Roorkiwal et al., 2018).

### Quantitative trait loci mapping for abiotic stresses in legumes

The heredity of abiotic stress resistance is a complex phenomenon, and QTL mapping is the most popular method of discovering QTL, as well as genetic and linkage mapping of genomic regions related to tolerance. QTL analysis allows researchers to examine the genetic structure of a characteristic. QTLs can identify genomic areas linked to the expression of the trait being researched (Kushwah et al., 2021). Strong linkage maps might be used for spatial replication of important genes. Because they are comprised of a sequence-tagged marker therefore these can also be used in functional genomics to analyze chromosomal arrangement and evolutionary purposes. Markers are successful in determining the QTLs for specific traits from various regions of chromosomes, for identifying genotypes against the different biotic stresses and multifunctional. QTLs were previously identified from the many legume crops by using linked markers (Luo et al., 2021; Zate, 2021). Advancements in phenomics and genomics now allowed us to better characterize the QTLs that influence a certain trait, admired as QTLome (Salvi and Tuberosa, 2015). Breeders have a responsibility to use QTLome's immense knowledge effectively because of its vastness. Improved QTL meta-analysis, estimations of QTL impact, and crop modeling will enable the QTLome to be used effectively against the abiotic stress of legumes. Several studies have been conducted to dissect QTLs in legumes crop for abiotic stresses. The identification of these QTLs against abiotic stress in various legumes are helpful for breeders during breeding activities. We have provided QTLs based studies in legumes against abiotic stress in Table 4.

**Table 4:** QTLs identified against abiotic stresses for various legumes crops.

**Crop**	**Abiotic stress**	**Associated marker (s)**	**Population type**	**Parental lines**	**Linkage group(s)/QTLs**	**References**
Chickpea	Salinity	SSR (135)	RILs	JG-62 × ICCV 2	LG3, LG6, and LG4	Vadez et al., 2012
	Salinity	SSRs (28) and SNPs (28)	RILs	JG 11 × ICCV 2	CaLG05 and 07	Pushpavalli et al., 2015
	Salinity	SSRs (150)	F2	Vignaluteola oblonga × Vignaluteola	LG1	Chankaew et al., 2014
	Heat	SNPs (271)	RILs	ICC 15,614 × ICC 4,567	CaLG05 and 06	Paul et al., 2018
	Cold	SNPs (747)	RILs	PI 489,777 and ICC 4,958	CTCa3.1 and 8.1	Mugabe et al., 2019
	Drought	SSRs (97)	RILs	ILC 3,279 × ILC 588	Q1-1 and Q3-1	Rehman et al., 2011
	Salinity	SSRs (150)	F2	Vignaluteola oblonga × Vignaluteola	LG1	Chankaew et al., 2014
Cowpea	Heat	SNPs (8)	RILs	IT82E-18 × CB27	Cht 5	Lucas et al., 2013
	Drought	AFLP (306)	RILs	CB46 × IT93K503-1	10 QTL (Dro)	Muchero et al., 2009
	Salinity	SSRs (32) and RFLPs (116)	F2:5	Tokyo × S-100	LG N	Lee et al., 2004
	Aluminum toxicity	SSRs, FLP, and AFLP (2,639)	RILs	Huaxia 3 × Zhonghuang 24	qAAC_04 and qRRE_04	Wang et al., 2019a
Soybean	Aluminum toxicity	DNA markers (14)	RILs	Forrest × Essex	LG F	Sharma et al., 2011
	Aluminum toxicity	SSRs (11)	RILs	NN1138-2 × KF No.1	LG B1	Korir et al., 2013
	Drought	SNPs (4,117)	RILs	Magellan × PI 567,731, PI 567,690 × Pana	Gm09, 05, 10, 06, 19, and 12	Ye et al., 2020
Pea	Salinity	SNPs (705)	RILs	Parafield × Parafield	Ps III and Ps VII	Leonforte et al., 2013
	Frost/cold	SNPs (258)	RILs	Terese × Champagne	LG5 and 6	Dumont et al., 2009
	Drought	SSRs (6) and SNPs (2)	RILs	cv Messire × P665	AA175, AB141, PsAAP2_SNP4, and A6	Iglesias-García et al., 2015
	Heat	SSRs (7)	F2	PDL-1 × E-153 and PDL-2 × JL-3	qHt_ps and qHt_ss	Singh et al., 2017a
Lentil	Frost/cold	AFLP (94), RAPD (56), and ISSR (106)	RILs	Precoz × WA8649090	LG4	Kahraman et al., 2004
	Drought	SNPs (220) and AFLPs (180)	RILs	ILL 5,888 × ILL 6,002	QRSAVII: 21.94 QSL12IV: 103.83, QSL22VII: 21.94, QSPADVIII: 72.15 and QDRWVII: 21.93	Idrissi et al., 2016
Faba bean	Frost/cold1	SNPs (5)	RILs	Bean Gottingen Winter and Bean Pure Line 4628	LG-01, LG-02, LG-03, LG-04, LG-08, and LG-10	Sallam et al., 2016
Mung Bean	Drought	SSRs (313)	RILs	ZL × VC2917	qMLA2A and qPH5A	Liu et al., 2017
Common Bean	Drought	AFLP (53), RAPD (2), SSRs (42), and SNPs (127)	RILs	BAT 881 × G21212	Pv01 and 08	Diaz et al., 2018

### Genome-wide association studies (GWAS) for abiotic stresses in legumes

Genome wide-association mapping is another beneficial tool to mitigate the constraints which provides the benefits from the historical meiosis of the diversity panel but may also offer better precision (Breria et al., 2020). In comparison to bi-parental mapping, association mapping is much more viable and cost-effective (Narayana and von Wettberg, 2020). Experimental architecture and statistical assessments in association mapping are continually changing to reduce the effects of influencing variables, reduce false positives, and regulate minor allele consequences. Due to genetic linkage and population structure, marker-trait relationships are muddled, creating a state of instability lacking real correlations. Numerous diagnostics or efficient statistical methods were developed to reduce false positives as well as minor allele QTL influences. Investigations of developmental genes including vernalization decreased height, and photoperiod response has been conducted at the exact genomic regions that are employed as a standard of comparison to integrate phenotypic variation with markers (Plewiński et al., 2020; Gondalia et al., 2022). These identified genes, being a strong genetic basis, can adjust the legumes plants under stress by changing heading time, plant height, and maturity time. In legumes, association mapping/GWAS is gaining popularity due to its capability to improve QTL discovery precision without investing additional resources in population growth. GWAS has made it possible to access functional genetic variations for salt tolerance traits in grain legume germplasms with greater accuracy and allelic richness (Hoyos-Villegas et al., 2017). Table 5 provide comprehensive information about the application of GWAS approach for the identification of genomic regions associated with abiotic stresses in legumes.

**Table 5:** Application of GWAS approach to identify the genomic regions associated with abiotic stresses in legumes.

**Crop**	**Abiotic stress**	**Markers used**	**Chromosomes/region/gene**	**References**
Common bean	Drought stress	SNPs (8,657) and SilicoDArT (3,213)	Chr. 6, 7, 10, and 11	Valdisser et al., 2020
	Drought tolerance	SNPs (3,724,159)	Chr. 01 and 06	Wu et al., 2021
	Drought tolerance	SNPs(3,832,340)	Chr. 10/*PvXIP1;2*	Wu et al., 2022
	Drought tolerance	SNPs (5,389)	Chr. 02, 03, 04, 06,09, 10, and 11	Dramadri et al., 2021
	Aluminum toxicity	SNPs (13,906)	Chr. 1, 4, 5, 6, 11	Ambachew and Blair, 2021
	Aluminum toxicity	SNPs (5,389)	Chr. 02, 04, 06, 07, 09, and 10	Njobvu et al., 2020
Mung bean	Salt and drought stress	SNPs	Chr.1, 7, 9, and 11	Breria et al., 2020
Alfalfa	Salt stress	SNPs (4,653)	All chromosomes except 2	Liu et al., 2017
*Phaseolus vulgaris* L.	Drought stress	SNPs	Pv01 and Pv02	Trapp et al., 2015
	Drought and heat stress	SNPs	Chr. 1, 2, 3, 11	Oladzad et al., 2019
	Flooding tolerance	SNP (~203 K)	Pv07 and Pv08	Soltani et al., 2018
	Heat stress	SNP (23,373)	Chr. 1–11	Assefa et al., 2019
*Glycine max* (L.)	Salinity stress	SNPs	Chr. 3	Patil et al., 2016
	Flooding tolerance	Multi-locus random-SNP	QTN	Yu et al., 2019
	Flooding tolerance	SNPs	Chr 03, 4, 07, 13, and 19	Wu et al., 2020
	Flooding tolerance	SNPs (34,718)	*Glyma.01G198000, Glyma.05G008000, and Glyma.08G348500*	Sharmin et al., 2021
	Drought tolerance	SNPs (12,316)	Chr. 01–15	Sertse et al., 2021
*Cicer arietinum* L.	Salinity stress	DArTseq markers (1,856)	Chr. Ca4 and Ca2	Ahmed et al., 2021
	Drought tolerance	SNPs (144,777)	Chr. 01–08	Li et al., 2018
*Vicia faba* L	Heat tolerances	SNPs (10,749)	Chr. 9 and Chr. 11	Maalouf et al., 2022
*Macrotyloma uniflorum*	Drought stress	SNPs (20,241)	–	Choudhary et al., 2022
*Camelina sativa*	Salt tolerance	SNPs (17)	Chr. 1, 7, and 20	Luo et al., 2020
*Pea*	Frost tolerance	SNP (11366)	LGI, LGII, LGIII, LGV, LGVI	Beji et al., 2020

### Genomic selection in legumes

With the development of model-based linkages with the widespread accessibility of genetic markers emerges a new concept called genomic selection which is developed to evaluate genotype pairing potential (Heffner et al., 2009). This method is used to overcome the limitations of map-based scientific studies of genetics, which only identify a few QTLs to explain the diversity in selected characteristics. Populations with much more allelic diversity of desired characteristics have a more accurate analysis of the QTL effect than a population that is strongly linked. Connection instability is regularly overstated in inter-mating individuals and it fades in subsequent meiotic activities (Nawaz and Chung, 2020). The genomic breeding potential is usually anticipated through genomic choice by altering marker-assisted selection using markers. The established model evaluated by genotypic and phenotypic facts of the research population will be applied to measure the phenotypic diversity of the selected respondent based solely on genetic makeup. This will increase yield potential in comparison to both QTL and phenotype trait selection (Shu et al., 2013; Sandhu et al., 2021). Statistical techniques are applied to create genomic selection models which depict the characteristics of many traits along with markers (Jannink et al., 2010). An optimum streamlined genome that is nonbiased assesses the genetic similarity between individuals based on molecular marker relatedness as well as evaluates the phenotypic efficiency (Nadaf et al., 2012). These models are near to the assessment of breeding value (BV) from heredity as well as physical competence of associated genetic makeup in a pedigree. According to statistical assessments, forward or mixed sort regression models can eliminate markers associated with different substantial impacts. Ridge regression also includes a penalized factor in markers design that is more than the statistically allowed number (Annicchiarico et al., 2017). The use of genomic selection (GS) in legumes began with the goal of enhancing yield and agronomic traits in soybeans. GBS was used to genotype 301 elite breeding lines, which were then phenotyped for grain yield in several localities (Jarquín et al., 2014). Several genomic selection studies have been conducted for legumes and some of them are given in Table 6.

**Table 6:** Genomic selection studies in legumes.

**Legume crop**	**Population size**	**Marker type**	**Traits**	*Genomic selection models	**References**
Soybean	301	GBS	Grain yield	G-BLUP model	Jarquín et al., 2014
	5,000	4,236 SNPs	Yield, protein content, and height	DualCNN, deepGS, RR-BLUP	Liu et al., 2019
	249	23,279 SNPs	Amino acid	RR-BLUP model	Qin et al., 2019
	5,600	4,600 SNPs	Yield	G-BLUP	Howard and Jarquin, 2019
Alfalfa	190	GBS (10,000 SNPs)	Single harvest biomass Total biomass	RR-BLUP model	Chapman, 2015
	278 (adapted to two different environments)	GBS	Dry matter yield	RR-BLUP model	Annicchiarico et al., 2017
Pea	372	331 SNP	Date of flowering Number of seeds per plant and Thousand seed weight	LASSO PLS SPLS Bayes A, Bayes B, and G-BLUP	Burstin et al., 2015
	339	9,824 SNPs (GenoPea 13.2 K SNP Array)	Date of flowering Number of seeds per plant Thousand seed weight	(kPLSR), LASSO, G-BLUP, Bayes A, and Bayes B	Tayeh et al., 2015
Chickpea	320	3,000 DArT markers	Seed yield 100 seed weight Days to 50% flowering, Days to maturity	RR-BLUP, kinship GAUSS, Bayes Cπ, Bayes B, Bayesian LASSO, Random Forest (RF)	Roorkiwal et al., 2016
	320	8,900 SNPs	Seed weight, harvest index and biomass	Reaction norm models	Roorkiwal et al., 2018
	132	144,777 SNPs	Seed number and grain yield	BL and BRR	Li et al., 2018
Groundnut	188	2,356 DArT markers	Days to flowering, seed weight, and pod yield	RR-BLUP, kinship GAUSS, Bayes Cπ, Bayes B, Baysian LASSO and RF	Pandey et al., 2014
	340	13,355 SNPs	Seed wight, yield and days to maturity	Reaction norm models	Pandey et al., 2020
	281	493 SNPs	Leaf let length, days to maturity and 100 seed weight	RR-BLUP	Akohoue et al., 2020
Common bean	**481**	**5,820 SNPs**	**Grain yield and days to maturity**	**G-BLUP**	Keller et al., 2020

## Transcriptome profiling for abiotic stress tolerance in legumes

Transcriptomics is a strong approach for quantifying gene expression along with the accurate image of a target cell or tissue. Transcriptomics properly identifies the gene regulatory pathway as well as candidate genes that are responsible for abiotic stress resilience in legumes and help in crop breeding (Afzal et al., 2020). Serial analysis of gene expression (SAGE) and microarrays might be used to determine extensive transcriptome information due to advanced technology like high-throughput analysis. Differential gene expression (DGEs) is usually evaluated by sequencing of nucleic acid (RNA-seq). A relatively newer invented technique known as digital gene expression (DGE) is used for the quantified gene expression measurement. RNA-seq is a low-cost, high-throughput sequencing technique for evaluating vast quantities of transcriptome data. This method has various advantages over microarray technology, including the fact that it should not need genomic data to create probe sets and can detect unique transcripts (Lowe et al., 2017). This method has been used in many studies to identify the gene regulatory process which is responsible for the tolerance to abiotic stress, particularly on pulse crops (Table 7). Using the NGS method, a transcriptome process has been generated under stressful circumstances like drought in soybean (Libault et al., 2010). The transcriptional alterations in drought-tolerant as well as drought-sensitive soybean cultivars have been documented using a comparative transcriptome process (Wang et al., 2018). Different varieties of common bean genotype which are vulnerable to biotic and abiotic conditions such as drought, aluminum toxicity, low phosphorous, and heat were evaluated for paternal polymorphism, functional and molecular genomic data association by single nucleotide polymorphisms (SNPs) markers, that was obtained from Sanger sequencing along with Illumina's GoldenGate method (Blair et al., 2013). Das et al. (2017) explained that the metabolomics monitoring of nitrogen or sugar metabolism and phytochemical metabolic activity are all important during water shortage situations in soybean. Singh et al. (2017b) identified potential genes involved in drought stress during lentil seedlings predicated on a transcriptome study, while Pandey et al. (2008) discovered dehydration-responsive proteins in chickpeas. Molina et al. (2008) detected 80,238 labels indicating 17,493 distinct transcripts in chickpea transcriptomes with drought conditions by employing SuperSAGE and deep SuperSAGE. The rapid generation of jasmonate into chickpea roots in drought situations was revealed by root transcriptome profiling of particularly oxylipin production genes. Future potential breeding initiatives may benefit from the use of RNA-seq to better comprehend the genes expressed under stress conditions (Dai et al., 2021).

**Table 7:** Transcriptome profiling of legumes crops under abiotic stress using RNA-Seq.

**Legumes crops**	**Abiotic stress**	**Plant parts**	**Seq. platform**	*Details	**References**
Chickpea	Drought	Roots and Shoots	Illumina HiSeq 2500	Identification of transcription factors linked with drought tolerance	Mahdavi Mashaki et al., 2018
		Root	Illumina HiSeq 2500	Identification of drought-resistant transcription factors Bhlh, C3H, NAC, AP2-EREBP, and MYB.	Shukla et al., 2006
		Leaf	Illumina HiSeq 3000	1,562 genes were differentially expressed in the tolerant genotype based on RNA extracted from leaf tissues. Genes that respond to drought were elevated in the tolerant genotype.	Badhan et al., 2018
	Salinity and Drought	Root apex	Roche 454 FLX	Under transcriptome profiling, miRNA-mediated post-transcriptional regulation of genes involved in lateral root development and re-patterning of root hair cells, as well as genes with a high affinity for K+ absorption. The root apex was used to dissect salt and water deprivation situations.	Khandal et al., 2017
Common bean	Drought	Leaf	Illumina GAIIx	During drought stress drought-sensitive genes were detected.	Wu et al., 2014
	Drought	Leaf and root	Illumina platforms (GAII and HiSeq 2000)	Data from transcriptomes showed novel genes associated with the drought stress response.	Confortin et al., 2017
	Salinity	Cotyledon, hypocotyl, and radicle	Illumina HiSeq 2500 PE 150	During the sprouting stage under salt stress, the role of zinc finger proteins (C3H) was discovered.	Zhang et al., 2020
		Root	Illumina HiSeq TM 2000	Transcriptome analysis revealed a total of 2,678 transcription factors, 441 of which were involved in salinity tolerance.	Hiz et al., 2014
Cowpea	Drought	Leaf	Illumina deep sequencing technology	There were just drought-responsive miRNAs identified.	Barrera-Figueroa et al., 2011
	Drought	Leaf		For drought-responsive genes, an SSH database (http://sshdb.bi.up.ac.za/, viewed on 3 August 2021) was generated.	Coetzer et al., 2010
	Cold (Chilling)	Pods	Illumina HiSeq 2500	Many sRNAs and miRNAs are implicated in the response to chilling, according to sRNAomic and transcriptome analyses.	Zuo et al., 2018
		Leaf	Illumina HiSeq 4000	During the vegetative and blooming stages, a total of 538 and 642 putative Transcription factors were found, respectively.	Khan et al., 2019a
Faba bean	Drought	Root	Illumina HiSeq 4000	New DEGs with altered expression during drought were discovered.	Alghamdi et al., 2018
	Salinity	Cotyledons	Illumina HiSeq 4000	A total of 1,410 salinity-responsive genes were discovered, with the salt-tolerant genotype showing considerable up-regulation of these genes.	Yang et al., 2020
Lentil	Drought	Leaf	Illumina HiSeq 2500	Drought-tolerant genotypes had more severe upregulation of genes involved in oxidation-reduction processes, TCA cycle, organ senescence, and stomatal conductance decrease than drought-sensitive genotypes.	Razzaq et al., 2021
	Heat	Leaf	Illumina HiSeq 2000	The cell wall and secondary metabolite pathways were both found to be significantly affected.	Singh et al., 2019
Mung bean	Desiccation	Seed	Illumina HiSeq 2500 with PE125	Many transcription factors (AP2, NAC, MYB, and methyltransferase and histone genes were discovered to be differently expressed.)	Tian et al., 2016

*AP2-EREBP, APETALA2/Ethylene-Responsive Element Binding Protein; bHLH, beta Helix Loop Helix; bZIP, beta Leucine Zipper; DEGs, Differentially expressed genes; HSPs, Heat shock proteins; LEA, Late embryogenesis associated; MADS, minichromosome maintenance factor1, agamous, deficiens, and serum response factor; miRNA, MicroRNA; MYB, myeloblastosis; NAC, NAM/ATAF1/CUC2; RNA, Ribonucleic acid; SSH, Suppression subtractive hybridization; sRNA, small RNA; TCA, Tricarboxylic acid cycle; TFs, Transcription factors.

## Metabolomics, proteomics, and ion-omics advances for abiotic stress in legumes

Abiotic stress has a significant impact on plants' metabolomes and proteomes, besides the change in genetic makeup and nucleotide sequences which are actively engaged in defense mechanisms against various stresses (Arbona et al., 2013). The proteome of an individual, which serves as a link between both the transcriptome and the metabolome more accurately represents the current level of proliferation and differentiation than Genetic markers. The transcriptome's assessment of cellular mRNA is not an exact representation of transcriptional activations (Min et al., 2019). The synthesis protein has played a role in cell signaling pathways and is implicated in stress adaption, stress repair, and also involved with other functions of the host plant. As a result, they aid the plant's recuperation from stress damage as well as make its survival much better in challenging situations (Hakeem et al., 2012). Conversely, metabolites, as opposed to mRNA or proteins, are a depiction of the transcriptional interactions that are involved in the regulation of gene expression throughout adverse conditions and have intimate linkages to the phenotype (Wienkoop et al., 2008). Metabolomics has been the most cross-functional of all systems biology and is present in many of the functions discussed (Arbona et al., 2013). Finally, detailed investigations proved that the metabolic networks participating in the growth and development of plants under a variety of environmental factors are critical for plant growth. Previously, metabolomics and proteomics were used to evaluate the molecular characteristics of legumes against abiotic stressors, as shown in Table 8. Ionomics is the science of a single tissue or the entire organism which encompasses the assessment of all elemental components in response to physiological functions or their alterations. Lahner et al. (2003) were the first who described an “ionome” as the metals, nonmetals, and metalloids present in an organism. Eventually, the terminology “ionome” was expanded to a “metallome” to relate to a collection of ecologically essential non-metals like nitrogen (N), sulfur (S), and phosphorus (P) (Salt et al., 2008). Ions play an important role in maintaining a plant's homeostasis under a variety of environmental situations. Ion transporters are also essential for the regular functioning of metabolic processes as well as managing stress. Particularly compared to various other grain crops, the ionomics technique has been widely employed in model legume plants (*Lotus japonicus*) and commercial legume crops including soybean (Chen et al., 2009). At the commercial level, the soybean genotypes with modified seed chemistry were found using this technique. Rapid innovations in ionomics have opened up new avenues for obtaining a precise description of the macro-and micronutrients or even the chemical compositions of legume grains in timely and cost-effective ways. Ionomics can be employed in this sense for attaining global food and nutritional security while also addressing the “hidden” starvation caused by micronutrient deficiencies (Hacisalihoglu and Settles, 2017; Ziegler et al., 2018). Finally, the ionome evidence can be used in experiments on the accessibility of micronutrients in fundamental pulses. Ionomics involves a thorough understanding of the transcriptional regulation concerned with homeostasis to assess the abiotic-stress-responsive ion transporters, genetic mutations compounds, and other constituents. Combining ionomics with other pan-omics tools, such as metabolomics and proteomics, will expand the possibilities for evaluating the impacts of abiotic stresses in legumes and their implementations helps in increasing legume productivity (Singh et al., 2021).

**Table 8:** Combating abiotic stresses in legumes using proteomics and metabolomics approaches.

**Crop**	**Abiotic stress**	**Omics approach**	**Details**	**References**
Chickpea	Drought	Proteomics	Potential resources for improving drought tolerance were identified.	Vessal et al., 2020
	Drought	Comparative proteomics	A total of 75 proteins were found to be differentially expressed in roots.	Gupta et al., 2020
	Drought	Comparative proteomics	MALDI-TOF/TOF-MS/MS analyses revealed 24 differently expressed proteins in leaves under drought stress.	Çevik et al., 2019
	Drought	Metabolomics	Effect of PGPRs under drought stress was identified using UPLC-HRMS analysis	Khan et al., 2019b
	Heat	Comparative proteomics	A total of 482 heat-responsive proteins were found to be engaged in heat stress tolerance.	Parankusam et al., 2017
	Salinity	Comparative proteomics	Various proteins were found to be engaged in salinity tolerance.	Arefian et al., 2019
	Drought	Metabolomics	Effect of PGPRs under drought stress was identified using UPLC-HRMS analysis	Khan et al., 2019b
Cowpea	Drought	Metabolomics	GC-TOF-MS profiling of primary metabolites and LC-DAD profiling of secondary metabolites under drought stress. Prolonged stress irrespective of the developmental stage affected the metabolome.	Goufo et al., 2017
Faba bean	Drought	Proteomics	Proteins including chitinase, Bet, and glutamate–glyoxylate aminotransferase were found to be upregulated in leaves under drought stress.	Li et al., 2018
	Salinity	Metabolomics	Molecules such as myo-inositol, allantoin, and glycerophosphoglycerol were found to be up-regulated in roots in response to salt stress.	Richter et al., 2019
	Drought and heat	Metabolomics	Upregulation of nitrogen and metabolism under combined heat and drought stress.	Das et al., 2017
Soybean	Salinity	Comparative metabolomics	A total of 47 different metabolites were found to be responsible for salt tolerance.	Li et al., 2017
	Aluminum	Comparative proteomics	MALDI TOF analysis revealed differential protein expression in roots under Al stress.	Duressa et al., 2011

## Phenomics prospective in legumes

The phenotype of an organism seems to be an observable biophysical property like appearance, behavior, and development (Pratap et al., 2019). Phenomics is a research area that involves high-throughput phenotyping analysis. Phenotype results from a complex combination of genetic capability among an organism and its environment (Deshmukh et al., 2014). Understanding any biological process needs precise phenotyping. A specific phenotype is often employed in plant and animal studies (like symptoms) to comprehend the biological condition, including disease, pest infestation, or physiological disorders. Genetic resources are employed for phenotype-based assessment of genetic markers, which arise as a result of technological advancements; known as “genetic symptoms.” The performance of genomics is determined by the consistency with which a genetic marker and phenotype are connected (Varshney et al., 2018). Plant breeding is using omics approaches to improve genetics to obtain an ideal phenotype that will provide a greater and more consistent yield under a variety of environmental situations. As a result, phenomics combined with certain other omics methods holds the most hope in plant breeding (Biswas et al., 2021). Abiotic stress tolerance seems to be a critical feature in terms of yield persistence and potential. Some traits which correlate to stress tolerance in legumes can be evaluated in a controlled condition at the time of sowing. The closing of stomata is one of the primary reasons for saline occurring in plants due to the osmotic effect of soluble compounds which are directly up taken by plant roots from groundwater. As a result, the photosynthesis process becomes slow. Therefore, an alternative measure can be exploited for stomatal responses and photosynthetic under osmotic tensity, which can also be stretched to legumes (Munns, 2010). Phenotyping concedes the imaging of characters of curiosity in real-time. NIRS (near-Infrared Spectroscopy) is one of the very dynamic techniques in advanced phenotyping study among plant breeding. For example, Jakubowski et al. (2017) chose various forage legume crops relying on their improved N_2_ fixation from the atmosphere by using this NIRS technique. The plants were evaluated based on the highest tissue N levels, which were proven to increase N_2_ fixation in the plant from the atmosphere. This depicts how phenotypic scanning would be used to assess and selected plants. The technique can be implemented to various legumes as well as more aspects.

## Genome editing of legume crops against abiotic stress

Multiple candidate genes which are associated with response to abiotic stress are being explored using genome editing methods. To date, numerous biotechnological tools are present to identify these genes. Somaclonal variations, tissue culture, marker-assisted breeding, mutagenesis, wide hybridization, double haploids, and genetic transformation are the main tools of genome editing (Aasim et al., 2018). Genetic transformation (GT) is a famous biotechnological technique used to address the biotic or abiotic stresses of legume plants. Yet, no meaningful progress has been made so far anyway. Kumar and Fladung (2002) explained the genetic transformation process and revealed that the AMT (Agrobacterium Meditated Transformation) is the most useful and powerful technique applied in legumes for genome editing followed by microprojectile bombardment (MP), direct gene transfer to protoplast (DGTP), and electroporation (EP).

### Genetic transformation in legumes

In the modern era, the insertion of exotic genes from wild plants to legumes and other important field crops through GT technology has become more significant (Somers et al., 2003). The reliability of transgenic germplasms for reproducing has been proven to be extremely low. Transgenic approaches aid in the production of more durable, genetically engineered germplasm with increased vigor and tolerance to different abiotic challenges, as well as enhanced crop productivity and quality (Chandra and Pental, 2003). The invention of transgenic tools has aided in the identification of mechanisms behind the transcriptional activation linked to abiotic stress resilience. The cultivation of genetically modified legume crops with better agronomic practices has been a great achievement (Aasim et al., 2013). Vadez et al. (2008) described that little progress has been made due to the complicated genetic pathways which are involved to produce crops tolerant to abiotic stress. Previously, a lot of studies revealed that genetically modified legume plants have shown more resilience to abiotic stressors such as salinity, drought, and metals (Atkinson and Urwin, 2012; Sita and Kumar, 2020).

### Genome editing in legume crops

To reduce food insecurity, many strategies are being used to build a sustainable agriculture system. Genome editing (GE) for crop enhancement is one such technology that has the potential to build a climate-resilient agriculture system on a worldwide scale (Liu et al., 2013; Abdelrahman et al., 2018). Plant breeding techniques have been significantly influenced by GE technology, including novel strategies for making rapid and precise modifications in crop genomes to defend plants from diverse threats and improve crop yields (Taranto et al., 2018). Site-specific endonucleases such as zinc-finger nucleases (ZFNs), transcription activator-like effector nucleases (TALENs), and CRISPR-Cas9 are employed in genome editing approaches (Zhu et al., 2017). There are many studies of genome editing that have been conducted on major legume crops.

Soybeans are rich in oil and protein, and an escalating demand requires genetic development with gene-editing tools to meet rising needs and cope with fluctuating climate (Bao et al., 2020). Curtin et al. (2011) targeted the green fluorescent protein (GFP) coding region in soybean with a ZFN array developed *via* context-sensitive selection strategies. This approach designed two independent ZFN pairs by deleting up to 71 base pairs on the target. Curtin et al. (2016) also focused on disturbing miRNA maturation and miRNA gene expression regulation. Accordingly, two ZFN pairs were designed to target Dicer-like 1a (DCL1a) and Dicer-like 1b (DCL1b) genes in soybean. Moreover, Curtin et al. (2018) generated a suite of combinatorial mutant plants using TALENs within the G. max Dicer-like2 gene by whole-genome sequencing. In CRISPR-Cas conditions, different sites of two endogenous soybean genes (GmFEI2 and GmSHR) were targeted by a transgene (bar) and six sgRNAs, and a sgRNA was designed that resulted in mutations in the targeted DNA of hairy roots (Cai et al., 2015). Di et al. (2019) CRISPR/Cas9 system was enhanced by highly active 5 U6 promoters targeting Glyma03g36470, Glyma14g04180, and Glyma06g136900 genes. Consequently, nucleotide insertion, deletion, and substitution mutations occurred. The following year, to target 102 candidate genes Bai et al. (2020) constructed 70 CRISPR-Cas9 vectors and obtained 407 T0 sgRNAs mutant lines with 59.2% mutagenesis frequency, and 35.6% lines carrying multiplex mutations. As a result, increased nodulation of gmric1/gmric2 double mutants and gmrdn1-1/1-2/1-3 triple mutant lines with decreased nodulation were detected.

*L. japonicus* a model organism for legume crops with similar features to *M. truncatula*, organizes determinate nodules, like soybean and cowpea. In *L. japonicus*, CRISPR/ Cas9 can perform SNF (symbiotic nitrogen fixation)-related gene mutations with hairy root transformation (Wang et al., 2016). Cai et al. (2018a) edited cytokinin receptor Lotus histidine kinaz I-interacting protein (LjCZF1) to disclose the process of cytokinin signaling regulation of rhizobia-legume symbiosis. They found mutant lines with reduced infection threads and nodules, indicating that LjCZF1 is a better regulator of nodulation. Later, CRISPR/Cas9 technology revealed that leghemoglobin (Lbs) in *L. japonicus* resulted in early nodule senescence (Wang et al., 2019b).

Chickpea (*C. arietinum*) is a commercially important crop worldwide, and gene-editing tools can be used to eliminate the problems in its production. Badhan et al. (2021) performed a study that targeted drought tolerance-associated genes, 4- coumarate ligase (4CL) and Reveille 7 (RVE7), for CRISPR/Cas9 editing in chickpea protoplast and the knock-out of the RVE7 gene showed high-efficiency editing *in vivo*. These results showed that CRISPR/Cas9 DNA-free gene editing can be used for genes associated with drought tolerance in chickpea by utilizing protoplast. To date, this was the first and only study that used CRISPR/Cas9 gene editing in chickpea.

Cowpea (*V. unguiculata L*.) Walp., with high nutrition content and health benefits, has tolerance to low rainfall, symbiotic nitrogen fixation (SNF) capability, and low fertilization requirements that make it commercially important (Ji et al., 2019; Che et al., 2021). Ji et al. (2019) targeted the SNF genes by CRISPR/ Cas9-mediated genome editing in non-inheritable mutated hairy roots in cowpea and found restricted nodule formation in the mutants with disrupted alleles. Following them, Juranić et al. (2020) used CRISPR/Cas9 gene editing and identified three cowpea meiosis genes; REC8 (encodes meiotic recombination protein), SPO11-1 (encodes SPO11 protein; an initiator of meiotic double-stranded breaks), and OSD1 (encodes Ophiostoma scytalone dehydratase protein promoting meiotic progression) to induce cowpea asexual seed formation. They found defects in meiosis due to biallelic mutations in exon 1 and exon 3 of the SPO11-1 gene. Recently, Che et al. (2021) also used CRISPR/Cas9 and noticed mutations at the target leading to inhibition of cowpea meiosis gene VuSPO11-1.

Lentils are a type of pulse crop that is consumed all over the world, being a good source of protein, minerals, carbohydrates, vitamins, dietary fiber, and secondary metabolites like phenolic compounds. Explants from various lentil tissues, such as shoot apices, epicotyls, nodal segments, embryo axes, cotyledonary nodes, and roots, have been explored for *in vitro* plant regeneration for genetic modification (Mahmoudian et al., 2002; Sarker et al., 2003; Akcay et al., 2009). As the number of shoots regenerated per explant greatly affect the transformation efficiency and success of CRISPR/Cas9-based gene editing, optimization of the protocol with an appropriate combination of mineral media and hormones is required in the near future. Gene disruption to improve lentils may be made simpler, less expensive, and more accurate by gene editing.

Mung bean is a warm-season, self-pollinated and diploid (2*n* = 2x = 22) legume crop with a small genome size (Parida et al., 1990; Nair et al., 2013; Kang et al., 2014). One of the first legumes to have a genetic linkage map was the mung bean in the early 1990s (Fatokun et al., 1992, 1993). In the past, molecular methods with an emphasis on yield, nutrient improvement, and disease resistance have been used to improve mung beans. These methods included random amplified polymorphic DNA (RAPD), restriction fragment length polymorphism (RFLP), SNP, and SSR markers (Fatokun et al., 1993; Chankaew et al., 2011; War et al., 2017). In mung bean breeding projects, CRISPR/Cas9 gene editing has a lot of potential. A symbiosis receptor-like kinase gene was recently successfully targeted using CRISPR/Cas9 gene editing to stop symbiotic nitrogen fixation in cowpea (V. unguiculata) (Ji et al., 2019). The achievement of CRISPR/Cas9 in a Vigna system shows that gene editing can be used for other species including mung bean. Quality attributes and disease resistance would be some of the initial targets for gene editing in mung beans. Developing mung bean varieties that can withstand changing weather patterns would help in the further expansion of mung bean cultivation worldwide.

Faba bean is a diploid (2*n* = 2x = 12) and one of the most important cool-season grain legumes with high protein content, antioxidants, and a rich source of fiber (Ray and Georges, 2010; Sudheesh et al., 2019). A reference genome of faba bean is still lacking; however, significant progress has been made in creating genetic and genomic resources to facilitate molecular breeding. Application of CRISPR/Cas gene editing is hampered by the lack of an annotated reference genome for the intricate faba bean genome, especially when designing specific gRNA-targeted genes of interest. To date, no CRISPR/Cas9 studies have been reported for this crop.

Genome editing, particularly CRISPR/Cas9 is an effective technology for abiotic stress tolerance in legumes, which is still rarely used. Similarly, there are limited studies in the literature on the implementation of genome editing for abiotic stress tolerance in other crops (Debbarma et al., 2019). In legumes, Soybean genome editing using the CRISPR/Cas9 system has been reported to be successful (Bao et al., 2019). For example, using the CRISPR/Cas9 approach, the soybean gene GmFT2a (FT-Flower Locus T) related to flowering time was knocked out, resulting in GmFT2a mutants with delayed flowering (Cai et al., 2018b). The roles of GmHsp90A2 (Heat shock protein 90s) in soybean have recently been studied using stable transgenic lines overexpressing GmHsp90A2 as well as mutant lines produced using the CRISPR/Cas9 genome editing technique (Huang et al., 2019). Hsp90s is a stable and abundant protein chaperone involved in the protective stress response, and overexpression of GmHsp90A2 in the *Arabidopsis thaliana* model resulted in greater tolerance to heat stress in a prior study (Xu et al., 2013). The CRISPR/Cas9 deletion of the endogenous soybean GmHsp90A2 gene was verified up to the T1 generation in the study of Huang et al. (2019). Although there are fewer scientific studies on using accurate and targeted genome editing for abiotic stress tolerance in Fabaceae can be found, these approaches have immense potential for future use in legume molecular breeding. Here, we compiled a list of reports on the application of transgenic tools in legumes to abiotic stress resilience (Table 9).

**Table 9:** Application of transgenic tools for genome editing in legumes for abiotic stresses.

**Legume crop**	**Performance of transgenic**	**Gene**	**Sources**	**Protein coded**	**Promoter used**	**References**
Mung bean	Salt and drought stress	Coda	*A. globiformis*	Choline oxidase A	CaMV35S	Baloda and Madanpotra, 2017
	Salt stress	gly I	*Brassica juncea*	Glyoxalate	CaMV35S and CmYLCV	Bhomkar et al., 2008
	Salinity and drought stress	ALDRXV4	–	Osmoprotection and detoxification	CaMV35S	Singh et al., 2016
*Phseolus vulgaris*	Drought stress	HVA1 and bar	*Hordeum vulgare*	Late embryogenesis protein	CaMV35S	Nguyen and Sticklen, 2012
*Medicago sativa*	Salt tolerance	GmDREB1	*G. max*	DRE-binding protein	*A. thaliana* RD29A	Jin et al., 2019
	Drought stress	WXP1	*Medicago truncatula*	AP2 domain	CaMV35S	Zhang et al., 2005
	Freezing stress	Fe-SOD	*Nicotiana plumbaginifolia*	Fe-superoxide Dismutase	CaMV35S	McKersie et al., 2000
	Aluminum toxicity stress	Malate dehydrogenase	*M. sativa*	Malate dehydrogenase	CaMV35S	Tesfaye et al., 2001
	Freezing stress	Mn-SOD	*N. plumbaginifolia*	Mn-superoxide dismutas	CaMV35S	Song et al., 2019
	Freezing stress	SOD	*P. sativum and N. plumbaginifolia*	Superoxide dismutase	CaMV35S	McKersie et al., 1993
*C. Arietinum*	Oxidative stress	Cod A	*Arthrobacter globiformis*	Choline oxidase A	CaMV35S	Sharmila et al., 2009
	Osmotic and drought stress	p5cs	*Vigna aconitifolia*	O1-pyrroline 5-carboxylate synthase	CaMV35S	Bhatnagar-Mathur et al., 2009
*Arachis hypogea*	Drought stress	Alfin1, PDH45, and PgHSF4	Alfaalfa and Pea	–	CaMV35S	Ramu et al., 2016
	Drought stress	DREB1A	*A. thaliana*	DRE-binding protein	*A. thaliana* RD29A	Mathur et al., 2014
	Drought stress	MuNAC4	*Macrotyloma uniflorum*	NAC	CaMV35S	Pandurangaiah et al., 2014
	Drought stress	DREB1A	*A. thaliana*	DRE-binding protein	*A. thaliana* RD29A	Vadez et al., 2013
	Drought and SALINITY stress	MuWRKY3	*Macrotyloma uniflorum*	WRKY	CaMV35S	Kiranmai et al., 2018
	Drought and salinity stress	AtHDG11	*A. thaliana*	Transcription factor	*A. thaliana* RD29A	Banavath et al., 2018
	Drought and salinity stress	SBASR-1	*S. chiata*	Abscisic acid stress	CaMV35S	Tiwari et al., 2015
	Drought and salinity stress	AtNAC2	*A. thaliana*	NAC	CaMV35S	Patil et al., 2014
	Drought and salinity stress	AtDREB2A and AtABF3	*A. thaliana*	DRE-binding protein	CaMV35S	Pruthvi et al., 2014
	Drought and salinity stress	SbVPPase	*Sorghum bicolor*	Vacuolar proton pyrophosphatase	CaMV35S	Puli et al., 2021
	Salinity stress	SbNHXLP	*S. bicolor*	Na1/H1 antiporterlikeprotein	CaMV35S	Kavi Kishor et al., 2018
	Salt stress	SbpAPX	*Salicornia brachiata*	Peroxisomalascorbate peroxidase	CaMV35S	Singh et al., 2014
	Salt stress	PDH45	*Pisumsativum*	DNA helicase 45	CaMV35S	Manjulatha et al., 2014
*Glycine max*	Drought and salinity stress	NTR1	*Bactris campestris*	Jasmonic acid	CaMV35S	Xue and Zhang, 2007

## Conclusion and future recommendations

Agriculture is dependent on the climate, and changes in climate are resulting in various stresses and causing significantl production losses each year. Legumes are multi-beneficial crops and can mitigate our food scarcity problems by providing high-quality food with low inputs. During the last two decades, significant progress has been made in utilizing state-of-the-art “OMICs” approaches to develop climate-smart legume cultivars. A huge number of legumes are conserved at the gene banks and also present in the small farmer's field and are yet to be characterized. Efforts should be done to characterize those unexplored legumes' genetic resources at both phenotypic and molecular levels to identify genetic variations that can be used for the development of improved cultivars. Nevertheless, a good number of efforts have been made to identify the genomic regions associated with abiotic stresses. However, fewer attempts have been made to validate the identified genomic region to speed up marker-assisted breeding. Therefore, it is a present-day need to develop KASP markers for speed breeding. Recent advancements in genomic resources for legumes have provided the basis to perform transformative breeding approaches like genomic selection and genome editing for crop improvement. However, these efforts are still few compared to cereals. It is very important to harness CRISPR/Cas9-based gene-editing technology for the targeted improvement of traits in legume crops.

## Author contributions

MN conceived the idea and perform editing to the manuscript. MN, NB, and MA collected the literature. AA, MTA, AS, HA, and TH contributed to writing the original draft of the manuscript. AA, MTA, HA, NB, K-HB, and TK designed the tables and figures. MN, FB, MA, AS, SD, TH, and TK reviewed and edited the manuscript. MN, FB, YC, NA, and MH contributed to addressing comments raised by the reviewers. MN, FB, and YC provided resources. All authors have read and approved the final version of the manuscript.

## Funding

The authors are very grateful to the Scientific Research Unit of Sivas University of Science and Technology for providing a research grant to MN through his project (Project Number: 2021-GENL-TBT-0003). Basic Science Research Program supported this research through the National Research Foundation of Korea (NRF), funded by the Ministry of Education (2019R1A6A1A11052070).

## Conflict of interest

The authors declare that the research was conducted in the absence of any commercial or financial relationships that could be construed as a potential conflict of interest.

## Publisher's note

All claims expressed in this article are solely those of the authors and do not necessarily represent those of their affiliated organizations, or those of the publisher, the editors and the reviewers. Any product that may be evaluated in this article, or claim that may be made by its manufacturer, is not guaranteed or endorsed by the publisher.
